# Effect of quality of bowel preparation on quality indicators of adenoma detection rates and colonoscopy completion rates

**DOI:** 10.1093/gastro/gov002

**Published:** 2015-02-12

**Authors:** Tarun Rai, Udayakumar Navaneethan, Tushar Gohel, Amareshwar Podugu, Prashanthi N. Thota, Ravi P. Kiran, Rocio Lopez, Madhusudhan R. Sanaka

**Affiliations:** Digestive Disease Institute, The Cleveland Clinic, Cleveland, OH, USA

**Keywords:** bowel preparation, adenoma detection rate, colonoscopy completion rate, quality indicators

## Abstract

**Background and aim:** Adequate bowel preparation is important for safe and effective colonoscopy. Quality indicators (QI) for colonoscopy include achieving at least 95% completion rate and an adenoma detection rate (ADR) of at least 25% in average-risk men and 15% in average-risk women aged over 50. Our aim was to investigate the impact of bowel preparation on ADR and colonoscopy completion rates.

**Methods:** This retrospective cohort study included patients who underwent colonoscopy between January 2008 and December 2009. The main outcome measurements were ADR and colonoscopy completion rates to the cecum.

**Results:** A total of 2519 patients was included; 1030 (41.0%) had excellent preparation, 1145 (45.5%) good-, 240 (9.5%) fair-, and 104 (4.1%) poor preparation. Colonoscopy completion rates were significantly lower in patients with poor or fair preparation (72.1% and 75.4%, respectively) than in those with good and excellent preparation (99.7% and 99.9%, respectively; *P < *0.001), and significantly lower than the QI of 95% (*P < *0.001). ADR in men and women combined was similar in all four grades of preparation (excellent, good, fair and poor) at 24.2% *vs. *26.8% *vs. *32.1% *vs. *22.1%, respectively; *P = *0.06. All the groups had ADR above the QI (25% for men and 15% for women) with evidence of significantly higher ADR in the women with excellent or good preparation and in men with excellent, good or fair preparation. On multivariate analysis, male gender was significantly associated with increased ADR (*P < *0.001), while the quality of bowel preparation did not influence ADR.

**Conclusions:** Patients with fair and poor standards of preparation have significantly lower colonoscopy completion rates than those with excellent and good preparation. However, there was no difference in ADR between the different grades of preparation.

## Introduction

Colon cancer is the third leading cause of diagnosed cancer and the second greatest cause of cancer death among both men and women in the USA [1]. Colonoscopy has become the standard screening tool for preventing colorectal cancer (CRC) by removing pre-cancerous adenomatous polyps. On the basis of large, population-based studies, several quality measures for colonoscopy have been established, such as adenoma detection rate (ADR) and colonoscopy completion rate. Consensus guidelines recommend some quality indicators (QI), including a minimum ADR of ≥25% in men and ≥15% in women [2, 3], colonoscopy completion rate measured by cecal intubation rate to be ≥95% [2, 4, 5] and colonoscopy withdrawal time of 6 minutes or more, in both men and women, to ensure safe and effective colonoscopy.

Interval cancers are reported after a colonoscopy and the majority of these are attributed to ineffective polypectomy [6], or to missing lesions, especially in the proximal colon [7, 8]. The ability to detect polyps is dependent on the quality of bowel preparation of the colon. Inadequate bowel preparation leads to decreased cecal intubation and inability to complete colonoscopy, and is hence believed to be a major contributor to ineffective polypectomy. But the study results for association between quality of bowel preparation and ADR have been mixed. One study found that only adenomas ≤9 mm were missed [9]; some studies found that there was a difference in both small and large adenomas [10–12], whereas in other studies no difference in ADR was noted, related to quality of bowel preparation [13]. No previous study had explored the relationship between quality of bowel preparation and completion of colonoscopy. Given the importance of this in routine clinical practice and the controversy over the role of bowel preparation on ADR, we used the large cohort who were undergoing screening colonoscopy at our institution to assess the relationship between quality of bowel preparation and both ADR and completion of colonoscopy in average-risk individuals over the age of 50.

## Methods

### Patients

This cohort study was approved by the Cleveland Clinic Institutional Review Board. Waiver of consent was obtained, due to its retrospective nature. Data was collected from the institution's electronic database. Endoscopists at our institution report all colonoscopies using a standard computerized endoscopy report generator. We retrieved this information covering the period from January 2008 to December 2009. Colonoscopy reports reviewed were from a total of 66 endoscopists, including 41 gastroenterologists, 15 colorectal surgeons, 8 general surgeons, and 2 primary care providers. A total of 2519 average-risk patients who met the inclusion criteria were included in the study.

### Inclusion and exclusion criteria

The inclusion criteria were age-appropriate screening colonoscopy in average-risk patients. The exclusion criteria were patients with (i) diagnosis of inflammatory bowel disease (IBD), familial adenomatous polyposis (FAP) or hereditary non-polyposis colorectal cancer, (ii) colonoscopy due to lower GI bleed or positive hemoccult test, (iii) work-up for iron deficiency anemia, (iv) chronic diarrhea, (v) previous history of colon resection or surgery, (vi) age less than 50 years, (vii) personal history of polyps and (viii) family history of polyps or colorectal cancer.

### Bowel preparation

All the patients who underwent colonoscopy had the procedure performed on an outpatient basis. Bowel preparation was done either with polyethylene glycol (PEG) or MoviPrep® (Salix Pharmaceuticals, Inc., Raleigh, NC, USA) (PEG-3350, sodium sulfate, sodium chloride, potassium chloride, sodium ascorbate and ascorbic acid for oral solution). The patients were given instructions for the use of bowel preparation solution.

### Diagnostic criteria

ADR was defined as percentage of colonoscopies with at least one adenoma detected. Proximal colon was defined as the colonic segment proximal to the splenic flexure. Distal colon was defined as colon distal to the splenic flexure. Colonoscopy completion was defined as completion of colonoscopy to the cecum.

Bowel preparation quality, recorded on electronic reports in our institution, is graded as a four-option scale, corresponding to the Aronchick scale [14], using the following ratings: 1 = excellent: small volume of clear liquid or more than 95% of surface visible; 2 = good: large volume of clear liquid covering 5–25% of the surface but more than 90% of the surface visible; 3 = fair: some semi-solid stool that could be sucked or washed away but more than 90% of surface visible; 4 = poor: semi-solid stool that could not be sucked or washed away and less than 90% of surface visible.

### Demographic and clinical variables

From the endoscopy reports, we collected demographic information (age, sex and race/ethnicity), procedural data (endoscopist, fellow participation, bowel preparation quality and sedation type), and endoscopic findings (polyp location, size and removal technique). Colonoscopy findings were retrieved, including indications, quality of preparation, polyp size, location, morphology and pathology. Endoscopists classified the bowel preparation quality as excellent, good, fair, or poor, based on the rating described above. Patients received moderate conscious sedation (opiate and/or benzodiazepine) or deep sedation (sedation administered by an anesthesiologist, with or without general anesthesia). Our institute’s electronic medical record system was used to collect pathology results associated with polyps. Polyp size was determined through both estimated size from the endoscopy report and pathology report: the larger of the two was recorded.

### Outcome measurements

The primary outcomes were the rates of detection of at least one adenoma and completion of colonoscopy in average-risk individuals. The secondary outcomes include the impact of bowel preparation on ADR and colonoscopy completion rates.

### Statistical analysis

Descriptive statistics were computed for all variables. These included means and standard deviations (SD) or medians and interquartile ranges [IQR] for continuous factors, and frequencies for categorical factors. Univariate analysis was performed to assess differences between the four preparation quality groups. Analysis of variance (ANOVA) or the non-parametric Kruskal-Wallis test were used to assess differences in continuous variables and Pearson’s chi-squared test was used for categorical factors; *ad* *hoc* pairwise comparisons were made using a significance criterion of 0.0008 (0.05/6) in order to account for multiple comparisons. In addition, a multivariate logistic regression analysis was performed to assess differences in ADR between the preparation quality groups, after adjusting for possible confounders such as gender and completion of procedure. The presence of at least one adenoma was modeled as the outcome with preparation quality, age, gender, sedation, complete procedure, type of scope used, previous colonoscopies and fellow involved as the independent variables. All analyses were performed using SAS version 9.2 software (The SAS Institute, Cary, NC) and R version 2.13.1 (The R Foundation for Statistical Computing, Vienna, Austria).

## Results

### Demographic and clinical characteristics

Out of 7357 patients screened, a total of 2519 patients who met the inclusion criteria were studied. The study cohort comprised 1030 (41.0%) with excellent preparation, 1145 (45.5%) with good preparation, 240 (9.5%) with fair preparation, and 104 (4.1%) with poor preparation. Their mean age was 59.4 ± 8.0 years and 1324 (52.6%) were men. The demographic and clinical characteristic of the patients is detailed in Table 1. Patients with poor preparation were found to be older than patients with excellent or good preparation (62.5 ± 9.2 *vs*. 59.3 ± 7.9 and 59.0 ± 7.9 years, respectively; *P < *0.001).

**Table 1.** Demographic and clinical characteristics

Factor	Quality of bowel preparation				*P*-value
	Excellent (*n = *1030)	Good (*n = *1145)	Fair (*n = *240)	Poor (*n = *104)	
Men	515 (50.0)	619 (54.1)	134 (55.8)	56 (53.8)	0.18
Age, years	59.3 ± 7.9^a^	59.0 ± 7.9^a^	60.2 ± 8.4	62.5 ± 9.2	*<0.001*
Conscious state^d^					0.62
Conscious sedation	1024 (99.5)	1139 (99.5)	240 (100.0)	104 (100.0)	
Monitored anesthesia care	5 (0.49)	6 (0.52)	0 (0.0)	0 (0.0)	
Prior colonoscopy done	72 (7.0)^c^	44 (3.8)	15 (6.3)	9 (8.7)	*0.006*
Complete procedure	1029 (99.9)^a^^b^	1141 (99.7)^a^^b^	181 (75.4)	75 (72.1)	*<0.001*
High-definition scope	266 (25.8)^c^	222 (19.4)^a^^b^	75 (31.3)	36 (34.6)	*<0.001*
Fellow involved	1017 (98.7)^c^	1104 (96.4)^b^	240 (100.0)	104 (100.0)	*<0.001*
Specialty of endoscopist					*<0.001*
Gastrointestinal	673 (65.3)^c^	635 (55.5)^a^^b^	180 (75.0)	81 (77.9)	
Colorectal	174 (16.9)	377 (32.9)	37 (15.4)	15 (14.4)	
Surgery	134 (13.0)	87 (7.6)	18 (7.5)	8 (7.7)	
Other	49 (4.8)	46 (4.0)	5 (2.1)	0 (0.0)	
Polyps	408 (39.6)^b^	504 (44.0)^a^	119 (49.6)^a^	30 (28.8)	*<0.001*
No. of polyps					*<0.001*
0	622 (60.4)^b^	641 (56.0)^a^	121 (50.4)^a^	74 (71.2)	
1	223 (21.7)	257 (22.4)	61 (25.4)	14 (13.5)	
2	106 (10.3)	136 (11.9)	26 (10.8)	9 (8.7)	
3+	79 (7.7)	111 (9.7)	32 (13.3)	7 (6.7)	
Distal polyps	299 (29.0)	362 (31.6)	87 (36.3)	23 (22.1)	*0.031*
No. of distal polyps					*0.03*
0	731 (71.0)	783 (68.4)	153 (63.8)	81 (77.9)	
1	195 (18.9)	214 (18.7)	60 (25.0)	16 (15.4)	
2	70 (6.8)	101 (8.8)	17 (7.1)	4 (3.8)	
3+	34 (3.3)	47 (4.1)	10 (4.2)	3 (2.9)	
Proximal polyps	195 (18.9)^b^	261 (22.8)	70 (29.2)	18 (17.3)	*0.002*
No. of proximal polyps					*0.002*
0	835 (81.1)^b^	884 (77.2)	170 (70.8)	86 (82.7)	
1	132 (12.8)	179 (15.6)	43 (17.9)	14 (13.5)	
2	44 (4.3)	51 (4.5)	19 (7.9)	1 (0.96)	
3+	19 (1.8)	31 (2.7)	8 (3.3)	3 (2.9)	
Adenomas	249 (24.2)	307 (26.8)	77 (32.1)	23 (22.1)	0.055
No. of adenomas					*0.035*
0	781 (75.8)^b^	838 (73.2)	163 (67.9)	81 (77.9)	
1	159 (15.4)	198 (17.3)	45 (18.8)	16 (15.4)	
2	67 (6.5)	68 (5.9)	18 (7.5)	5 (4.8)	
3+	23 (2.2)	41 (3.6)	14 (5.8)	2 (1.9)	
Distal adenomas	137 (13.3)	166 (14.5)	44 (18.3)	10 (9.6)	0.11
No. of distal adenomas					0.13
0	893 (86.7)	979 (85.5)	196 (81.7)	94 (90.4)	
1	106 (10.3)	124 (10.8)	36 (15.0)	6 (5.8)	
2	26 (2.5)	35 (3.1)	6 (2.5)	3 (2.9)	
3+	5 (0.49)	7 (0.61)	2 (0.83)	1 (0.96)	
Proximal adenomas	148 (14.4)	194 (16.9)	51 (21.3)	16 (15.4)	0.056
No. of proximal adenomas					*0.038*
0	882 (85.6)^b^	951 (83.1)	189 (78.8)	88 (84.6)	
1	107 (10.4)	143 (12.5)	30 (12.5)	13 (12.5)	
2	34 (3.3)	29 (2.5)	15 (6.3)	2 (1.9)	
3+	7 (0.68)	22 (1.9)	6 (2.5)	1 (0.96)	

Values presented as mean ± SD with ANOVA or *n* (%) with Kruskal-Wallis test for number of polyps or adenomas and Pearson's chi-squared test otherwise.

A significance level of 0.008 was used for pairwise *ad hoc* comparisons:

^a^Significantly different from poor

^b^Significantly different from fair

^c^Significantly different from good.

^d^Data were not available for one subject with excellent bowel preparation.

### Colonoscopy completion rate

**Figure 1.**
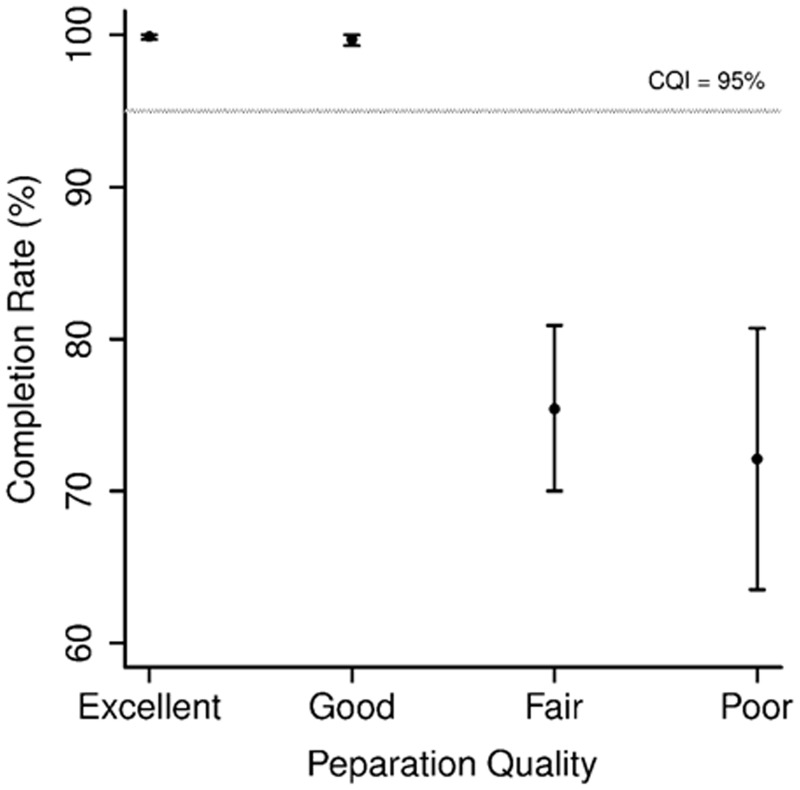
Colonoscopy completion rates and preparation quality.

Colonoscopy was completed in 2426 (96.3%) patients. Subjects with poor or fair preparation had lower colonoscopy completion rates than those with excellent and good preparation (72.1% *vs*. 75.4% *vs*. 99.9% *vs*. 99.7%, respectively; *P < *0.001) (Table 1). Completion rates in patients with fair or poor preparation were significantly lower than the QI of 95% (*P < *0.001) (Figure 1).

### Polyp and adenoma detection rates

Polyps and adenomas were detected in 1061 (42.1%) and 656 (26.0%) patients, respectively. Proximal polyps and adenomas were detected in 544 (21.6%) and 409 (16.2%), respectively, whereas distal polyps and adenomas were detected in 771 (30.6%) and 357 (14.2%) patients, respectively. Overall, there was a trend towards significance in the difference between preparation quality and ADR (*P = *0.055). Patients with fair preparation were more likely than those with either excellent or poor preparation to be found with at least one polyp (49.6% *vs*. 39.6% *vs*. 28.8%, respectively; *P < *0.001). In both the proximal and distal colon, polyps were more likely to be detected in patients with fair preparation than in the other groups. Similarly, ADR was significantly higher in the fair preparation group than in those with excellent preparation (32.1% *vs*. 24.2%, respectively; *P = *0.007). This was mainly due to a higher rate of detection of proximal adenomas in patients with fair preparation (21.3% *vs*. 14.4%, respectively; *P = *0.005). There was no difference in distal ADR between the fair preparation group and the others (*P = *0.11).

### Adenoma detection rate by gender

**Figure 2.**
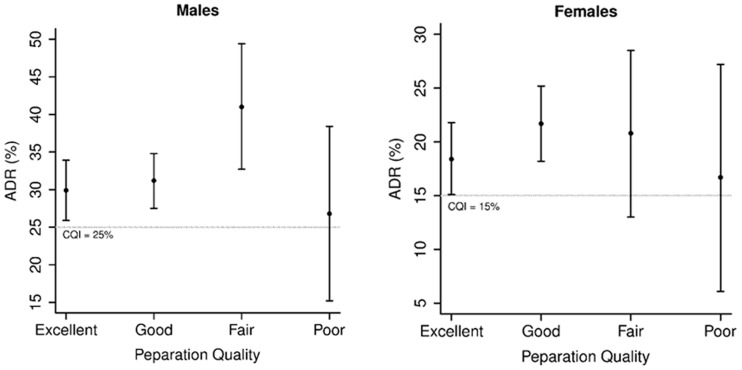
Adenoma detection rate in men and women, based on quality of bowel preparation (The circle represents the completion rate and the whiskers extend to the 95% lower and upper confidence limits).

There was no evidence to suggest that ADR was associated with preparation quality in either men and women (*P = *0.076 and *P = *0.56, respectively) (Table 2 and Table 3). All the groups had ADR above the QI (25% for men and 15% for women) with evidence of significantly higher ADR in the women who had excellent or good preparation and in men with excellent, good or fair preparation (Figure 2).

**Table 2.** Adenoma detection rate in men

	Quality of bowel preparation				*P*-value
	Excellent (*n = *515)	Good (*n = *619)	Fair (*n = *134)	Poor (*n = *56)	
ADR (%)	154 (29.9)	193 (31.2)	55 (41.0)	15 (26.8)	0.076
Distal ADR (%)	83 (16.1)	100 (16.2)	32 (23.9)	7 (12.5)	0.12
Proximal ADR (%)	15 (26.8)	127 (20.5)	38 (28.4)	11 (19.6)	0.092

ADR = adenoma detection rate.

**Table 3.** Adenoma detection rate in women

	Quality of bowel preparation				*P*-value
	Excellent (*n = *515)	Good (*n = *526)	Fair (*n = *106)	Poor (*n = *48)	
ADR (%)	95 (18.4)	114 (21.7)	22 (20.8)	8 (16.7)	0.56
Distal ADR (%)	54 (10.5)	66 (12.5)	12 (11.3)	3 (6.3)	0.5
Proximal ADR (%)	53 (10.3)	67 (12.7)	13 (6.3)	5 (10.4)	0.092

ADR = adenoma detection rate.

### Adenoma detection rate by experience

The length of endoscopists' experience ranged between 1 and 34 years, with a median of 11 [5–24] years. We arbitrarily divided them into junior (≤5 years work experience) and senior endoscopists (>5 years work experience). In average-risk subjects with excellent or good preparation, 67% of procedures were performed by senior endoscopists and 33% were performed by juniors. ADR was 27% in the junior group, compared to 25% in the senior group (*P = *0.38).

### Multivariate analysis

We did a multivariate analysis for ADR after adjusting for age, gender, sedation, and complete procedure, type of scope used, previous colonoscopies and fellow involvement. The quality of preparation was not found to be significantly associated with ADR. Male gender was significantly associated with increased ADR (odds ratio [OR] 1.8; 95% confidence interval [CI] 1.5–2.2; *P < *0.001) (Table 4).

**Table 4.** Multivariate logistic regression analysis for adenoma detection rate

Variables	Odds ratio (95% CI)	*P*-value
Preparation quality (excellent vs. poor)	1.3 (0.75–2.1)	0.39
Preparation quality (good vs. poor)	1.4 (0.85–2.4)	0.18
Preparation quality (fair vs. poor)	1.7 (1.00–3.0)	0.052
Age (every 5 year increase)	1.05 (1.00–1.1)	0.064
Male	1.8 (1.5–2.2)	<0.001
No previous colonoscopy	1.3 (0.85–2.0)	0.23
High definition scope	1.2 (0.99–1.5)	0.063
Sedation	1.2 (0.31–4.6)	0.8
Complete colonoscopy	0.86 (0.51–1.4)	0.57
Fellow involved? (No *vs. *Yes)	1.2 (0.65–2.1)	0.6

## Discussion

Inadequate preparation quality for colonoscopy has been associated with a lower ADR if polyps are less than 10 mm in size whereas, in other studies, no difference in ADR with quality of bowel preparation was reported. Our study showed that ADR was not affected by quality of bowel preparation after adjusting for age, gender, fellow involvement and completed procedures. There was a trend towards a higher ADR in patients with fair preparation than in those with poor preparation but this did not reach statistical significance. Male gender was associated with higher ADR on multivariate analysis.

ADR—which is defined as the percentage of procedures per colonoscopist in which at least one adenoma is detected—has been considered to be a strong quality measure for effective colonoscopy [2, 3, 15–17]. In our study, male gender was associated with increase in ADR, similarly to previous studies. Some of the other risk factors for higher ADR, such as ethnicity, diabetes, and smoking, were equitably distributed in all the patient groups in our study. Patients with a family history of CRC and large adenomas have higher prevalence of adenomas on subsequent colonoscopies but, in our study, we excluded all the high-risk patients and others undergoing colonoscopy for any indication other than screening, so these factors could not be measured [18–21].

We found that patients with fair preparation produced the highest ADR and polyp detection rate. As reported in studies, the ADR in average-risk individuals undergoing screening colonoscopy is highly variable (14.9–37.5%), whereas advanced adenoma and large adenoma detection rate varied between 5.2–9.7% and 3.4–8.5% [22, 23]. In our study group, overall ADR was 22.1% in patients with poor preparation and 32.1% with fair preparation; thus, at our centers, the skilled endoscopists kept the ADR consistently above the recommendations. It is possible that during procedures for fair preparation, colonoscopists might had invested more inspection time during colonoscopy withdrawal and hence enhanced the ADR. Colonoscopy withdrawal time was not recorded and it is a limitation of this retrospective study. It would have been interesting to study whether an increase in withdrawal time was responsible for the increase in ADR of our fair preparation patients.

Our study has several clinical implications. It asserts that quality of bowel preparation influences the colonoscopic completion rate. Colonoscopy completion rate was 75.4% and 72.1%, respectively, for patients with fair and poor preparation, compared to 96.3% and 99.4% in patients with excellent and good preparation. Also, we observed that fair bowel preparation quality does not decrease the detection rate of adenomas of any size and does not necessitate reduction in colonoscopy surveillance interval. Although the overall ADR was not significantly lower in patients with poor preparation, a larger sample size of patients might have detected a difference in patients with poor preparation when compared with fair, good or excellent preparation.

There are some limitations to our study. There might have been a referral bias, as patients were recruited from a tertiary care center. The skill levels of endoscopists at our tertiary care center may not be generally applicable to community practice. Another potential limitation of the study is involvement of fellows in the colonoscopies, but there was no difference in the fellow participation in any of the studied groups. So, if there were any difference, it would have been equally distributed across all the groups. Another limitation of our study is that all the patients underwent colonoscopy in an office-based setting. It has been reported that bowel preparation quality varies between the inpatient *vs*. outpatient scenarios. Also, we did not collect information on body mass index, the relationship to ADR of which is controversial. Also, colonoscopy withdrawal time was not recorded, which may have influenced ADR. In our study cohort, excellent and good standards of preparation were noted in 41.0% and 45.5% patients, respectively. The possible reasons for better colonoscopy preparation in our cohort could be (i) the inclusion of only patients undergoing screening colonoscopy, (ii) mean age lower than other studies and (iii) an office-based setting. Also we don’t have data for aborted procedures due to poor or fair preparation. Additionally, none of the patients with poor or fair preparation subsequently had CT colonography or barium enema, so that data is not available. In our practice, these patients are typically brought for surveillance colonoscopy earlier than at the recommended surveillance intervals (for example, surveillance colonoscopy is done after 1 year instead of 3 years)

In conclusion, our study showed that excellent-to-good bowel preparation quality is associated with an increased rate of colonoscopy completion, but does not significantly influence ADR. Fair bowel preparation quality does not decrease ADR and does not necessitate a reduction in colonoscopy surveillance interval. We need further prospective studies to confirm these findings in an even larger cohort.

*Conflict of interest statement*: none declared.
